# Expanding the role of clinical pharmacists on interdisciplinary primary care teams for chronic pain and opioid management

**DOI:** 10.1186/s12875-018-0783-9

**Published:** 2018-07-03

**Authors:** Karleen F. Giannitrapani, Peter A. Glassman, Derek Vang, Jeremiah C. McKelvey, R. Thomas Day, Steven K. Dobscha, Karl A. Lorenz

**Affiliations:** 1 VA Palo Alto Health Care System, Center for Innovation to Implementation (Ci2i), Menlo Park, CA 94025 USA; 2 VA Greater Los Angeles Health Care System, Center for the Study of Healthcare Innovation, Implementation, and Policy (CSHIIP), Los Angeles, CA 90073 USA; 3 David Geffen School of Medicine, University of California, Los Angeles, 10945 Le Conte Ave, Los Angeles, CA 90024 USA; 4 VA Minneapolis Center for Chronic Disease Outcomes Research (CCDOR), 5445 Minnehaha Avenue South, Minneapolis, MN 55417 USA; 5 VA Northern California Health Care System, 10535 Hospital Way, Mather, CA 95655 USA; 6 VA Portland Health Care System, Center to Improve Veteran Involvement in Care (CIVIC), 3710 SW US Veterans Hospital Rd, Portland, OR 97239 USA; 7 Department of Psychiatry, Oregon Health and Science University, 3181 SW Sam Jackson Park RD, Portland, OR 97239 USA; 8 Stanford Medical School, Palo Alto, CA 94305 USA; 9 RAND Corporation, 1776 Main Street, Santa Monica, CA 90401 USA

**Keywords:** Pain, Pain management, Clinical pharmacists, Team based care, Interdisciplinary teams, Qualitative research

## Abstract

**Background:**

Facilitating appropriate and safe prescribing of opioid medications for chronic pain management in primary care is a pressing public health concern. Interdisciplinary team-based models of primary care are exploring the expansion of clinical pharmacist roles to support disease management for chronic conditions, e.g. pain. Our study aims to 1) identify roles clinical pharmacists can assume in primary care team based chronic pain care processes and 2) understand the barriers to assuming these expanded roles.

**Methods:**

*Setting*: Veterans Health Administration (VA) has implemented an interdisciplinary team-based model for primary care which includes clinical pharmacists. *Design*: We employed an inductive two part qualitative approach including focus groups and semi-structured interviews with key informants. *Participants:* 60 members of VA primary care teams in two states participated in nine preliminary interdisciplinary focus groups where a semi-structured interview guide elucidated provider experiences with screening for and managing chronic pain. To follow up on emergent themes relating to clinical pharmacist roles, an additional 14 primary care providers and clinical pharmacists were interviewed individually. We evaluated focus group and interview transcripts using the method of constant comparison and produced mutually agreed upon themes.

**Results:**

Clinical pharmacists were identified by primary care providers as playing a central role with the ongoing management of opioid therapy including review of the state prescription drug monitoring program, managing laboratory screening, providing medication education, promoting naloxone use, and opioid tapering. Specific barriers to clinical pharmacists role expansion around pain care include: limitations of scopes of practice, insufficient institutional support (low staffing, dedicated time, insufficient training, lack of interdisciplinary leadership support), and challenges and opportunities for disseminating clinical pharmacists’ expanded roles.

**Conclusions:**

Expanding the role of the clinical pharmacist to collaborate with providers around primary care based chronic pain management is a promising strategy for improving pain management on an interdisciplinary primary care team. However, expanded roles have to be balanced with competing responsibilities relating to other conditions. Interdisciplinary leadership is needed to facilitate training, resources, adequate staffing, as well as to prepare both clinical pharmacists and the providers they support, about expanded clinical pharmacists’ scopes of practice and capabilities.

## Background

Pain is a complex condition impacted by various biological, psychological, and social factors and is usually a high treatment priority for patients seeking care [1, 2]. Due to its complexity, chronic pain can be incredibly difficult to manage. Improving processes of care for chronic pain is a public health priority [3]. This is due to both the high prevalence of chronic pain diagnoses as well as the risks associated with inadequate management [4]. Since a majority of chronic pain is managed in primary care settings, primary care teams are increasingly overburdened by and not fully equipped to manage all of the complex, competing demands of patients with pain [5, 6]. With expanded patient panel sizes (the number of patients assigned to a specific provider) and limited appointment times, clinicians face prioritizing individual demands of specific medical conditions over other patient concerns.

These complex challenges can be particularly problematic in the Veteran’s Health Administration (VA) where providers are working with populations that experience high rates of chronic disease, including pain and other comorbid conditions [7]. The VA is the largest integrated health care system in the United Sates. VA cares for over nine million Veterans and is comprised of 170 medical centers and 1063 outpatient clinics [8]. In 2010, the VA implemented an interdisciplinary team-based model of primary care called patient-aligned care teams (PACTs). These teams include primary care providers (PCPs), nurses, and administrative staff, and are supported by other clinicians including licensed clinical pharmacists [9–12]. Expanding roles of clinicians such as advanced practice nurses and clinical pharmacists, to include top of license tasks such as prescribing, is conceptualized as central to improving chronic disease management while minimizing the burden on individual PCPs.

One strategy being explored to support chronic disease management involves embedding clinical pharmacists in primary care to facilitate patient education, supplemental patient interaction, and population management activities [11, 13–16]. Population management comprises reviewing the entire population of patients and proactively identifying risks; risk of inappropriate opioid use for pain management is an important public health concern. Some of the expanded clinical responsibilities a clinical pharmacist can assume around pain care include: ongoing reassessment, monitoring, and management of opioid therapy in conjunction with medication renewal, review of state prescription drug monitoring programs, and medication education. Some clinical pharmacists are specializing in pain management to support teams with complex pain patients, opioid risk management, opioid education, opioid titration, opioid screening, and naloxone distribution [15]. When clinical pharmacists are involved in an interdisciplinary team, clinics have reported a decrease of burden on PCPs and improved patient satisfaction [17].

To inform future implementation efforts we queried all providers involved in primary care based pain management to better understand clinical pharmacists’ current and potential roles with pain management. Further, the clinical pharmacists we interviewed included several layers of pharmacy leadership, which helped us explore system level barriers to assuming expanded roles. Existing literature suggests that expanding pharmacist’s clinical roles for chronic pain care may minimize adverse effects patients experience and thus improve patient care [18]. Our study aims to 1) identify roles clinical pharmacists can assume in primary care team based chronic pain care processes and 2) understand the barriers to assuming these expanded roles.

## Methods

### Overview

The data for this qualitative analysis was collected as part of the Effective Screening for Pain (ESP) study, a mixed methods analysis of pain screening, assessment, and management methods [19]. All study procedures were approved by the VA central Institutional Review Board (IRB Project ID 13–08). Under ESP we had multiple waves of primary data collection. Verbal consent to participate and record all focus groups and interviews was obtained at the beginning of each session.

### Setting

This study was conducted in the context of the VA. The VA relied on the patient centered medical home model (PCMH) to provide out-patient primary care. The VA’s version of PCMH is called patient aligned care teams (PACT). Under PACT, interdisciplinary providers work together to provide continuous, coordinated, high quality care. PACT patients are assigned to a specific interdisciplinary care team (including a primary care provider, registered nurse, licensed practical nurse, and clerk). These small interdisciplinary teams are supported by other providers such as behavioral and mental health providers, social workers, and licensed clinical pharmacists. Clinical pharmacists are those who participate in prescribing, patient education, population management activities, and possibly academic detailing or education to other providers on evidence based prescribing practices.

### Wave one data

In wave one, we conducted nine focus groups including a total of 60 providers working on interdisciplinary primary care teams in two large VA Medical Centers in California and Oregon, as well as associated community based outpatient clinics [20–22]. Interdisciplinary team members invited to participate in the focus groups included PCPs, registered nurses, licensed practical nurses, clerks, psychologists, social workers and clinical pharmacists. Focus groups occurred between 2013 and 2014 and lasted approximately one hour.

### Focus group analysis

Trained facilitators used a semi-structured interview guide to elucidate provider experiences with screening, assessment, and management of chronic pain. All focus groups were audio recorded, professionally transcribed, and transcripts were cleaned to remove identifying information. All analyses were conducted using qualitative analytic software ATLAS.ti. [23]. An initial code list was developed and iterated via the dual coding of two transcripts. The final code list was then systematically applied to every transcript. After primary coding, secondary coders reviewed each transcript for inconsistencies. Team meetings fostered consensus for code development and facilitated resolutions for coding discrepancies. ESP investigators evaluated transcribed interviews using the method of constant comparison [24] and produced mutually agreed upon themes. The method of constant comparison continued until we reached theoretical saturation on each theme. Presentation of results to PACT team primary care providers served as a member check and confirmed content validity. Expansion of clinical pharmacist roles emerged as a supportive factor to PACT pain management. As a result of this early finding we conducted further investigation through a second wave of interviews.

### Wave two data

The second wave of interviews consisted of individual semi-structured interviews with 14 key participants. The participants included: PCPs (general internists and nurse practitioners) as well as multiple types of clinical pharmacists (primary care, mental health, pain management, and pharmacy leadership). These semi-structured interviews occurred at Portland and Palo Alto in 2016. The interview guild included specific probes about how clinical pharmacists are involved in chronic pain management (see Table 1). Key stakeholders were identified using a snowball sampling approach [25], and we relied heavily on referrals for recruitment. Snowball sampling represented the best method we had to access the clinical pharmacists, as we did not have a master list of people in this role. Through introductions we were able to recruit a meaningful number that we may not have have been able to access via cold calling or emails.

**Table 1:** Focus Group and Interview Guide Questions

**Wave One Focus group questions** Who in your opinion is the most appropriate person to screen for pain and why? Who in your opinion is the most appropriate person to assess pain and why? Other than the finding that the patient has pain, what information about a patient’s pain is most useful to you in: a) preparing to assess pain b) actually assessing pain c) guiding your treatment/management plan for pain What are the roles of different staff members [PCP, RN, LVN, Clerk, Mental health, Pharmacist etc...] in getting pain information? Assessing pain? Managing pain? **Wave Two Semi-structured interview probes relating to pharmacist role expansion** How are pharmacists involved in primary care chronic pain management currently? How could their role expand? What are the barriers to role expansion? Would they need additional training or licenses? How does the pharmacist interact with the rest of the team about pain? For what types of conditions and concerns are they involved? What is the role of the academic detailer relating to pain medications? Do you have any thoughts about how we could improve pharmacist directed pain management of ‘at risk’ Veterans? What do you think about pharmacists taking on responsibility for monitoring and providing medications to such Veterans? Do you anticipate any challenges for pharmacists taking on that role? Are there others on the team who would be better equipped to take it on? Who (Nurses, Social workers, etc.)?	

### Wave two analysis

Informed by grounded theory [24], investigators [KG and DV] then used an open coding approach on the wave two interview transcripts to identify emergent themes. This method was selected to help us divide the textual data into conceptual components. Specifically, through iteration and consensus the entire investigator team reviewed all quotes associated with barriers and characterized emergent themes around barriers to the clinical pharmacists’ role expansion. This process continued until theoretical saturation was reached on each barrier. After the interviews were completed, one clinical pharmacist interviewee [JM] joined the investigator team during the analytic phase to provide expert guidance on contextualizing the results.

## Results

Respondents included 60 focus group participants in nine focus groups and 14 semi-structured interview respondents. Below we present first the results of the focus groups which explain the roles a clinical pharmacist can assume, second, we present results from the induvial interviews which cluster into three themes.

### Focus group results

The focus groups with different providers revealed substantial support across professions for an enhanced role of clinical pharmacists. PACT providers advocated for pharmasists to assume responsibility in the team process.

*“Overall if by the time I saw a patient they had been pre-screened or processed and behind the scenes somebody in pharmacy… had run the CURES reports [CURES: California’s Controlled Substance Utilization Review and Evaluation System] and arranged for the interval of tox screens [urine toxicology screening] … [and] were aware of any escalations in terms of early refill-if that information was available... [Then] I didn’t have to worry about it, that’d be helpful.” [PCP]*

Additionally, PCPs felt having the clinical pharmacists involved in refill management for patients on long term opioid therapy may offer a team strategy to minimize the risk of drug abuse.

*“If the pharmacy believes that they're not due for them, they will not give it [opioid pain medication] to them. Because you have some patients that they just got the pain medication today, come back the following day.... Because it was lost or they robbed their house. Of all the valuables, it's only the Vicodin that was stolen? It's very common… Our pharmacy department, they are very smart, too. So they tell them [patient], “Okay. Then come back every week.” And if they lose it again, “Then come back every day.” Instead of giving them the whole supply.” [PCP]*

PCPs also described including clinical pharmacists on the team as particularly useful for reconciling prescriptions from providers in multiple departments. In cases where acute pain, dental pain for example, combines with chronic pain, clinical pharmacist involvement can prevent patients exhibiting drug seeking behavior from accessing inappropriate quantities of opioid medications.

*“We've recently had a patient— complaining he was in pain. But the first thing he said was, “I'm not a drug addict, I have a family of four, I'm gainfully employed, I go to school,” … he was placed on Tramadol… He took the Tramadol [home]… He came back a week later; he [should] still have more Tramadol, [but] claimed he hadn't used it. He said he had a [new] dental problem, went up to Dental, requested something stronger. He had an extraction; he was ordered Tylenol #3. He took that, but he had gotten the Vicodin the three days before… he came back … Requesting more Vicodin. He also went to the pharmacist, and the pharmacist said, “You can't get it. It's not due.” [PCP]*

PCPs also highlighted the valuable contribution clinical pharmacist run clinics make in coordinating care with pain and substance abuse specialists.

*“For the chronic pain patients… they [clinical pharmacists] have the chronic or renewal clinics for pain medications and they actually do the assessments there… those clinics are pharmacist run, but they work very closely with both the actual pain clinic and the substance abuse clinics.” [PCP]*

*“So if the substance abuser has issues, they can actually go easily right into the pain clinic and if a pain clinic patient has a problem they go right easily into the substance treatment program and then the [clinical pharmacist run] renewal clinic does a lot of monitoring-they monitor the tox screens, they monitor the CURES reports, they monitor the pain levels if something needs to be adjusted.” [PCP]*

### Interview results

Three core themes were identified: limitations of scopes of practice, insufficient institutional support, and challenges and opportunities for disseminating clinical pharmacists’ expanded roles. The quotes we present below come from the clinical pharmacist interviews and these themes were also present in the PCP interviews.

### Theme 1: Limitations of scopes of practice

#### 1.1 Including pain management in a scope of practice

Some clinical pharmacists do not have a local scope of practice for pain management.

*“Currently my scope is only for the disease states I mentioned earlier [diabetes, dyslipidemia, hypertension or poly-pharmacy] and I don’t have a scope for pain.” [Clinical Pharmacist]*

*“We’ve tried to get some of the PACT pharmacists to help [with checking CURES report]; but, like I said, that pushback -not written within our scope- has been there.” [Pharmacy Leadership]*

#### Variation in scopes of practice relating to prescribing

Some clinical pharmacists were not scoped to prescribe controlled substances.

*“We [clinical pharmacists] can’t order the [controlled] medications. It just seems like something that would be kind of [helpful] in terms of time-wise and provider-wise” [Clinical Pharmacist]*

In some states clinical pharmacists can prescribe controlled substances, but providers may be unaware of clinical pharmacists’ scopes of practices.

*I met with a new provider the other day for addiction treatment services and was explaining my role as a pharmacist, and a lot of them just aren’t even sure that we have scopes of practice, that we actually see patients and prescribe. [Clinical Pharmacist]*

### Theme 2: Insufficient institutional support

#### Low staffing

Staffing levels pose a challenge for clinical pharmacists who wish to spend time doing additional training for having pain in their scope of practice.

*“Another problem is… the staffing… in order to take on additional training, then I would have to be taken away from my responsibilities in order to get the hours in to get the scope [to work on pain].” [Clinical Pharmacist]*

Insufficient local staffing may limit the time a clinical pharmacist has to address both chronic disease and opioid management.

*“I don't think we have the manpower to…deal with the chronic diseases—diabetes, hypertension, and lipids—and also troubleshoot the [opioid] medication. So don't have much time to deal with this pain thing.” [Clinical Pharmacist]*

#### Dedicated time

Clinical pharmacists consistently reported that lack of time was a barrier to taking on additional tasks such as managing pain medication and reporting.

*“For CURES [reporting] I don’t think that would be the lack of training [to take on that work]. I think that’s just maybe the time. They say that there’s a time limitation.” [Clinical Pharmacist]*

Clinical pharmacists identified that given their limited time, they would benefit from guidance around where their support would be best directed.

*“We know that when it comes to coag [anticoagulation] management and lipid management that they’re [PACT clinical pharmacists] not needed as much as they were, so there is time freeing up…what other disease states does the health care system want them to help with, whether it’s heart failure, COPD, pain. Where does pain…fall in that?” [Clinical Pharmacist]*

Some clinical pharmacists report that managing an assortment of medical conditions can make it difficult to become more involved with pain care.

*“Right now we are responsible for… the anticoag portion… It’s not something that I could just defer [to work on pain]… now we decentralized anticoagulation, so I’ll just follow all the Warfarin patients who belong to my providers… it’s a little bit difficult because… I try to schedule it as best as I can, but you just can’t really control when patients come in or when new patients go to the ER with a new blood clot. We do have set clinic hours for pharm care, like, when we do see diabetics but, other than that, I just have to fill in all my anticoags whenever I can.” [Clinical Pharmacist]*

#### Insufficient training

Some clinical pharmacists reported that they did not think their experiences and skills were adequate to work with pain management.

*“There's not much courses or a lecture on pain management…also, I don't have that much experience [with pain] in terms of clinic to kind of learn from my experience.” [Clinical Pharmacist]*

Clinical Pharmacists also reported limited training opportunities in pain management.

*“I [clinical pharmacist with pain specialty training] would assume it’s [pain management] beyond what their [PACT clinical pharmacists] current training is and that they would need education.” [Clinical Pharmacist]*

#### Lack of interdisciplinary leadership support

Support from leadership can facilitate implementing interdisciplinary team-based approaches to pain management.

*“It’s been several things… Great leadership. So our associate chief of primary care, as well as our nursing leadership and myself [clinical pharmacist] and primary care behavioral health, there’s four of us that get together. We put education together and we go on the road and we meet with everybody on a regular basis, and we just start piecing things together and we would identify who on the teams could help.” [Clinical Pharmacist]*

### Theme 3: Challenges and opportunities for disseminating clinical pharmacists’ expanded roles

#### Perceived capabilities

Clinical pharmacists believe some PCPs may have limited awareness of their capabilities.

*“I had stated at a meeting that we were piloting this program and then I had sent out an email, but it still seemed like the providers weren’t familiar with what we [clinical pharmacists] could do and what our involvement could be, so I guess that dissemination of education to the providers about what we could do [would be helpful].” [Clinical Pharmacist]*

Clinical pharmacists believe some PCPs do not know what clinical pharmacists can take on and have preconceived ideas as to what their roles are and can be.

*“I met with a new provider the other day for addiction treatment services and was explaining my role as a pharmacist, and a lot of them just aren’t even sure that we have scopes of practice, that we actually see patients and prescribe. I think a lot of providers might still be stuck in the idea that we’re still behind the counter in the pharmacy, actually dispensing the medications. So yeah, maybe more clarity on the different types of pharmacists and their actual roles and how we can collaborate together.” [Clinical Pharmacist]*

#### Self-advocate

Clinical pharmacists may have to self-advocate and remind PCPs of their capabilities.

*“ I’ve met with the majority of them [PCPs] in either staff meetings or one-on-one, and I’ve provided them with my contact information so whether it’s Instant Messaging, GUI, Outlook or calling me. Some of them attach me on a note, but I pretty much always try to say, “If you have any issues,” and even because I’m not a pain specialist, that’s not my area of expertise, but I try to resolve whatever barrier and seeing how can I assist? That’s just me kind of putting my face out there and trying to let them know that this resource is available.” [Clinical Pharmacist]*

#### Promoting awareness of the referral process

Clinical pharmacists highlight the need to make PCPs aware of the referral process to involve clinical pharmacists for pain medication management.

*“Making sure that they’re [PCPs] aware of the referral process to either have the pharmacist completes an e-consult or a chart review with recommendations or they can even refer patients to physically see the pain pharmacist in clinic.” [Clinical Pharmacist]*

## Discussion

The implementation of expanded roles for clinical pharmacists offers a central strategy to addressing the expanding need for primary care provision in the context of an impending physician workforce shortage. On the other hand, role expansions are complex undertakings. They require dedicated resources, training, and leadership support to successfully transition the roles of clinical pharmacists and surrounding PCPs with whom they share care for complex patients. The PCMH model highlights the ideal of “sharing the care” [26]. Explicitly “sharing the care” for chronic pain patients with embedded clinical pharmacists may ultimately improve patient satisfaction with care and outcomes while reducing PCP burnout associated with being alone in dealing with complex patient concerns.

In this qualitative study we set out to characterize interdisciplinary provider perspectives of how clinical pharmacists have several opportunities to fulfill enhanced roles in relation to chronic pain management in interdisciplinary primary care teams. We enumerate multiple barriers clinical pharmacists face. The strategies that may address emergent barriers included expanding primary care clinical pharmacist training so that they had a better understanding of pain as well as ensuring the team had access to a clinical pharmacist with pain expertise. Both PCPs and clinical pharmacists alike indicated the importance of having a pain trained clinical pharmacist, other providers could consult.

Given how quickly clinical pharmacy practice is evolving, it is not surprising that clinical pharmacists experience a number of barriers to expanding roles around chronic pain care. To understand pharmacist role expansion it is important to consider the historical distinctions between medicine and pharmacy practices. Our results indicate it is crucial to prepare for educating physicians about potential top of license tasks for clinical pharmacists as these may challenge current pre-conceived understandings of clinical pharmacist capabilities [27]. The barriers we identify in this study, namely low external awareness of clinical pharmacist capabilities, capacity, and prescribing authority, echo findings in other current studies [28, 29]. To overcome low awareness, interdisciplinary dialogue about clinical pharmacist roles is essential.

A central tenant of implementation science is the belief that if we understand and characterize individual barriers, we can develop targeted implementation strategies to address them [30]. In addition to training, resources, and staffing which indicate health system level strategies, the issue of how best to prioritize PCP and clinical pharmacist time for addressing chronic pain requires further consideration. Additionally, decentralization of care for some conditions (e.g. all clinical pharmacists cover a few anticoagulation patients each) versus use of clinical pharmacist dedicated clinic time for a specific condition (e.g. pain) can cause tension around clinical pharmacists’ workload distribution.

What we learned from the provider focus groups is pain is a condition where having multiple diverse providers involved can add value, particularly in regard to patient safety and monitoring activities. Known mitigation strategies of opioid overuse include regular monitoring and reassessment to provide opportunities to minimize risks. Periodic reassessment allows the opportunity for tapering or discontinuing opioids [3]. Our providers indicated that clinical pharmacists can clearly play a key role in this monitoring process. In addition to having technical expertise, clinical pharmacists added value to the team by giving PCPs a way to say no about prescribing high doses to patients. Simply having someone available representing the hospital policies or guidance on safe prescribing, was supportive to primary care providers trying to navigate difficult conversations with drug seeking patients.

Additional strategies that have been called for to reduce inappropriate opioid prescribing include updated protocols and clinical guidelines [31]. In many states clinical pharmacists can prescribe controlled substances and could take on the role of prescribing per protocol. Clinical pharmacist led, academic detailing campaigns have been demonstrated to reduce high-dose opioid prescribing [32]. Clinical pharmacists scoped to deal with pain may also have the ability to support patients through tapering and medication changes.

At present, few medical schools offer sufficient training on addiction and pain management [33]. Consequently, the average PCP may be underequipped to deal with substance seeking patients with pain in primary care. This is of concern as much of the prescribing for chronic pain is occurring in primary care [5]. Having clinical pharmacists on the interdisciplinary team to help navigate complex situations and minimize risk of addiction in chronic pain patients, could represent part of a solution.

Our findings should be considered in the context of the following limitations. First our study was conducted within the VA health care system alone. The VA, however, while not generalizable to other settings does have a population with a high prevalence of pain and an extensive history of addressing chronic pain in the primary care setting [34]. This makes the VA a strong setting in which to investigate pain practice processes. Secondly, this study represents the sub analysis of a greater study meaning that all of the methods are not specifically tailored for this inquiry. For example, the initial focus group data was collected for broader purposes. We do, however, think that the benefits of hypothesis generation (i.e. exploring the role of clinical pharmacists) outweigh the limitations of being unable to identify what specific team members were included. Third, although we conducted a limited number of focus groups, sites varied by location (rural, suburban, and urban), academic status, and size, allowing us to describe the perspectives of providers based in multiple environments. Another major limitation is that fundamentally, we do not know the exact roles of participants in the focus groups. We sought participation from all members of interdisciplinary pact teams, including clinical pharmacists, but do not know if clinical pharmacists were among those who participated. This approach was supported by the IRB. We were able to encourage broad participation of the PACT team members, allowing them to be honest and critical, because we did not collect any information on who they were.

Due to this ambiguity in the focus groups, one of the main reasons we conducted wave two of interviews was to explicitly capture the perspectives of clinical pharmacists on emergent themes. We interviewed providers as well and their comments confirm the perspectives of the clinical pharmacists. We chose to include only clinical pharmacists’ quotes in the wave two results section of the paper because they were the most descriptive. Though our sample of clinical pharmacists is relatively small, it represents the majority of clinical pharmacists at a single VA region as well as additional clinical pharmacists from a second geographic region. We would also note that in this study, we do not capture the perspectives of patients; this can be an important focus of future work.

The two potential sources of bias that may impact our results are that we included an interviewee as an author and that we employed a snowball sampling approach to identify clinical pharmacists. We invited a pharmasist investigator [JM] to participate on the manuscript after data collection was completed and after initial thematic analysis was drawn on the results. We chose to do this after collecting the data when it became apparent that we needed a clinical pharmacist expert to comment on our results with full depth of understanding. Further, we appreciate that snowball sampling has some inherent limitations in that it may attract like-minded individuals. However, it is the best method we had to access the clinical pharmacists. We did not have a master list of people in this role, so we requested introductions. Through this, we were able to recruit a meaningful number that we may not have gotten via cold calling or emails.

## Conclusions

These findings indicate that both providers and clinical pharmacists see an importance to expand clinical pharmacist roles in supporting management of complex pain patients. The potential benefits include reduced burdens on physicians and better guideline concordant opioid based pain care. Implementation barriers to their role expansion are not insignificant, but with appropriate targeted strategies, can be addressed. Roles and scopes of practice need to be clarified in advance. Primary care providers who work with clinical pharmacists need to then be made aware of clinical pharmacist scopes and capabilities. Interdisciplinary collaboration and communication between the medicine and pharmacy services will be essential to successful clinical pharmacist role expansion and shared team prioritization.
